# Real Time Breath Analysis Using Portable Gas Chromatography for Adult Asthma Phenotypes

**DOI:** 10.3390/metabo11050265

**Published:** 2021-04-23

**Authors:** Ruchi Sharma, Wenzhe Zang, Menglian Zhou, Nicole Schafer, Lesa A. Begley, Yvonne J. Huang, Xudong Fan

**Affiliations:** 1 Department of Biomedical Engineering, University of Michigan, Ann Arbor, MI 48109, USA; rusharma@umich.edu (R.S.); wenzhez@umich.edu (W.Z.); sharonml@umich.edu (M.Z.); 2 Division of Pulmonary and Critical Care Medicine, Department of Internal Medicine, University of Michigan, Ann Arbor, MI 48109, USA; nischafe@med.umich.edu (N.S.); labegley@med.umich.edu (L.A.B.)

**Keywords:** asthma, exhaled breath, portable gas chromatography, precision medicine

## Abstract

Asthma is heterogeneous but accessible biomarkers to distinguish relevant phenotypes remain lacking, particularly in non-Type 2 (T2)-high asthma. Moreover, common clinical characteristics in both T2-high and T2-low asthma (e.g., atopy, obesity, inhaled steroid use) may confound interpretation of putative biomarkers and of underlying biology. This study aimed to identify volatile organic compounds (VOCs) in exhaled breath that distinguish not only asthmatic and non-asthmatic subjects, but also atopic non-asthmatic controls and also by variables that reflect clinical differences among asthmatic adults. A total of 73 participants (30 asthma, eight atopic non-asthma, and 35 non-asthma/non-atopic subjects) were recruited for this pilot study. A total of 79 breath samples were analyzed in real-time using an automated portable gas chromatography (GC) device developed in-house. GC-mass spectrometry was also used to identify the VOCs in breath. Machine learning, linear discriminant analysis, and principal component analysis were used to identify the biomarkers. Our results show that the portable GC was able to complete breath analysis in 30 min. A set of nine biomarkers distinguished asthma and non-asthma/non-atopic subjects, while sets of two and of four biomarkers, respectively, further distinguished asthmatic from atopic controls, and between atopic and non-atopic controls. Additional unique biomarkers were identified that discriminate subjects by blood eosinophil levels, obese status, inhaled corticosteroid treatment, and also acute upper respiratory illnesses within asthmatic groups. Our work demonstrates that breath VOC profiling can be a clinically accessible tool for asthma diagnosis and phenotyping. A portable GC system is a viable option for rapid assessment in asthma.

## 1. Introduction

Asthma is a chronic inflammatory airway disease characterized by reversible airflow obstruction and episodic symptoms of wheezing and shortness of breath. However, asthma is clinically heterogeneous and while many phenotypes have been described, the mechanisms for most remain poorly understood. Type 2 (T2) -high asthma, linked to allergic inflammation, is the best understood endophenotype, defined by elevated Type 2 immune responses (e.g., eosinophilia and increased exhaled nitric oxide, etc.) and a better response to treatments like inhaled corticosteroids (ICS) and biologics targeting Type 2 cytokines [1]. In contrast, T2-low asthma represents a constellation of other phenotypes characterized by diminished or absent Type 2 inflammation, although atopy may still co-exist. Non-eosinophilic asthma may characterize up to 50% of asthmatic adults based on sputum eosinophil assessment [2,3]. Further understanding of T2-low asthma and identifying relevant biomarkers that inform underlying biology is of great interest; there currently are no treatments that target specific phenotypes of T2-low asthma.

Non-invasive approaches to investigate asthma biology are attractive because they decrease burden on research participants and may lead to the identification of clinically useful and deployable biomarkers. Breath analysis has increasingly been used to identify discriminatory patterns of exhaled compounds associated with asthma and other airway diseases [4,5]. To date, measurement of exhaled nitric oxide (FeNO) is the only such biomarker validated for clinical use [6] and is largely reflective of Type 2 inflammation, correlating with greater atopy and allergic inflammation [7]. Applications of electronic nose (eNose) technologies have demonstrated their ability to distinguish asthmatic from healthy subjects, as well as predict some clinical features including atopic status [8], circulating inflammatory patterns [9], and loss of asthma control [10]. However, eNose identifies composite signature patterns without direct ascertainment of the contributory chemical compounds. Identifying the specific exhaled metabolites associated with particular features of asthma would offer insights into potential biology contributing to that phenotype for further study. For example, a recent large study used gas chromatography-mass spectrometry (GC-MS) to identify specific exhaled compounds that distinguish eosinophilic from neutrophilic asthma, as defined by sputum cell counts [11]. More such studies are needed to elucidate exhaled metabolites that may serve as surrogate markers for other clinically important asthma phenotypes and also in different populations to inform clinical validity.

In this pilot study, we aimed to address some of these gaps by exploring in our U.S.-based adult cohort whether exhaled breath metabolites, measured by our portable GC system are capable to distinguish asthmatic from non-asthmatic/non-atopic and atopic non-asthmatic (atopic control) subjects. We also hypothesized that exhaled breath metabolites would discriminate subjects by blood eosinophil levels, obese status, and asthmatics on inhaled corticosteroid (ICS) treatments or experiencing an upper respiratory illness within asthmatic groups. With the detection, identification, and statistical analysis of the exhaled breath VOCs, we demonstrate the application of a portable GC system as a potential aid to asthma clinical diagnosis and therapeutic monitoring.

## 2. Results

### 2.1. Clinical Cohort

Asthmatic and non-asthmatic subjects were enrolled in a prospective observational study at the University of Michigan (CAARS; NCT02887911; clinicaltrials.gov (accessed on 26 March 2021), Bethesda, MD, USA) and provided written informed consent to participate in a longitudinal component of the study (MICROMAAP) entailing subsequent quarterly visits for a year. The study protocols were approved by the University of Michigan Institutional Review Board (HUM00097163 and HUM00136068). Subjects underwent detailed baseline assessments as previously described [12], including asthma and allergy history questionnaires and lung function testing (spirometry, methacholine challenge, and bronchodilator reversibility). Asthma diagnosis was confirmed by spirometry with positive methacholine challenge and/or bronchodilator reversibility, performed according to the American Thoracic Society/European Respiratory Society guidelines [13,14]. Exclusion criteria included significant smoking history (>10 pack-years) and acute lower respiratory illness, asthma exacerbation and/or systemic antibiotic use within 8 weeks of the baseline study visit. Blood was collected for complete blood count with differential cell analysis and determination of atopic sensitization to common respiratory allergens (specific IgE to 16 aeroallergens; Phadia ImmunoCAP). Presence of at least one positive specific IgE on this panel was considered evidence of atopy. Induced sputum was collected by inhalation of 3% saline for 12 min and used to determine sputum inflammatory cell counts. As summarized in Table 1, a total of 73 participants contributed 79 total breath samples. These included 30 asthma, 8 atopic non-asthma, and 35 non-asthma/non-atopic subjects evaluated between June 2018 and February 2020. All breath samples from MICROMAAP subjects were collected during study visits during the morning hours; study subjects had fasted overnight except for taking medications with water or using their prescribed inhaled therapies. Some exhaled air samples from control subjects (non-asthma/non-atopic) were collected from colleagues (33 breath samples) after informing them about the study and obtaining verbal consent. Three asthma patients who later developed upper respiratory illness and one asthma patient who took ICS treatment were respectively sampled twice over the course of regular study visits that occurred quarterly or in the setting of subsequent upper respiratory illness. For the latter scenario, asthma subjects returned for a study visit within one week of symptom onset for additional sample and data collection.

**Figure 1:**
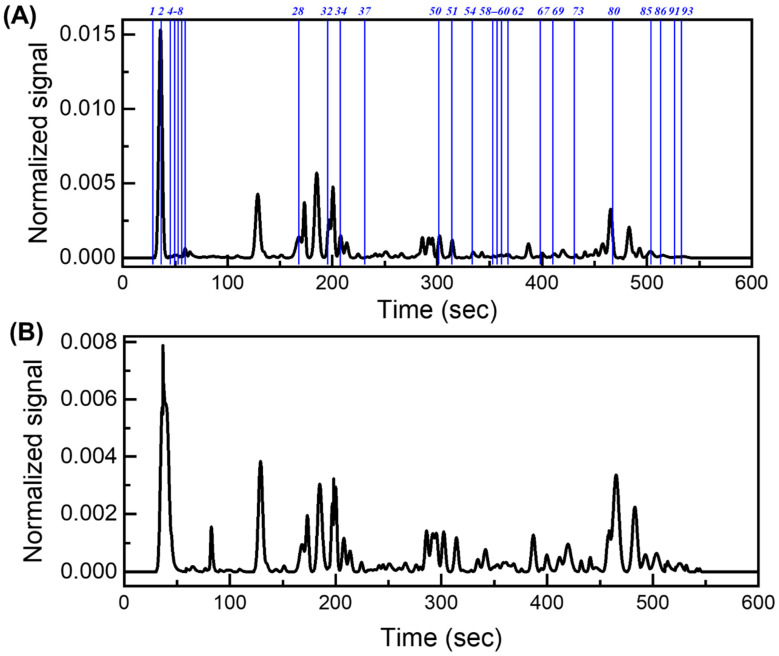
Representative GC chromatogram from an asthma patient (**A**) and a non-asthma/non-atopic (**B**). The blue lines and numbers mark the peak positions of the identified biomarkers listed in Table 2 and Table 3.

A total of 79 breath chromatograms were analyzed. Detailed description of the portable GC device and its operation can be found in Section 4, and Section S1 in the Supplementary Materials. After chromatogram pre-processing (see Section 4 and Section S2 in the Supplementary Materials), approximately 90 peaks can be detected in each breath chromatogram (Figure 1). Collectively, there were a total of 103 different peaks in the 79 chromatograms, although some of the 103 peaks could contain co-eluted VOCs. Finally, through machine learning, linear discriminant analysis (LDA), and principal component analysis (PCA) were used for biomarker selection and statistical analyses (see details in Section S3 in the Supplementary Materials).

### 2.2. Biomarkers to Distinguish Asthma and Non-Asthma

**Figure 2:**
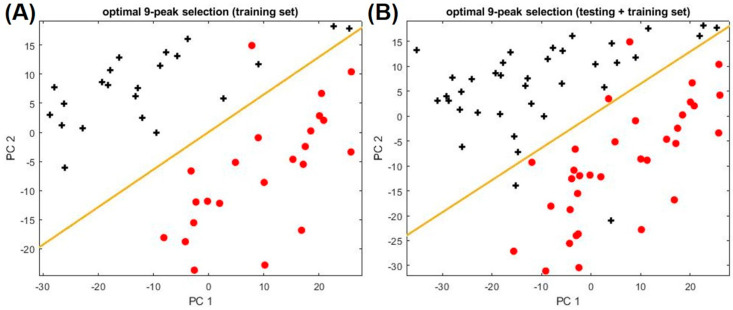
PCA plots using the optimal 9-peak subset (9 biomarkers) for distinguishing asthma from non-asthma/non-atopic subjects. (**A**) Training set. (**B**) Training set plus testing set. Asthma and non-asthma/non-atopic are denoted as red circles and black crosses, respectively. The yellow line marks the position of the boundary. The peak IDs and their chemical names of the nine biomarkers can be found in Table 2.

Not all peaks may be relevant to asthma, since some peaks may be from normal metabolic activities, other conditions that a patient may have, or exogenous factors (indoor air background, smoking, and use of consumer products, etc.) [15]. Therefore, it is critical to determine which subset of the peaks is most responsible for the differences observed between asthma and non-asthma groups. For selecting the optimal subset of peaks (i.e., biomarkers), 45 chromatograms from asthma and non-asthma/non-atopic were used as the training set, whereas the remaining 26 chromatograms were used as the testing set. As detailed in Section S3 of the Supplementary Materials, optimal peak subset (peak ID: 7, 32, 50, 51, 69, 73, 80, 85, 93 listed in Table 2) is identified through LDA, which yields the maximum classification accuracy of 94.4% and the maximum boundary distance to distinguish asthma and non-asthma. Those biomarkers are mainly from alkane families (see Table 3), some of which are same as or similar to biomarkers previously reported, such as Compound **32** (Heptane, 2,4-dimethyl), Compound **50** (Heptane, 2,2,4-trimethyl), Compound **51** (Octane, 3,3-dimethyl), Compound **69** (Heptane, 2,3,5-trimethyl-), Compound **73** (Decane, 2,4,6-trimethyl), and Compound **93** (Decane, 2,6,6-trimethyl) [16,17,18,19,20,21,22,23]. The details of the biomarker discovery process can be found in Section S3 of the Supplementary Materials. Figure 2 shows the PC plot of the training and the combined (training and testing) set. The corresponding statistics are given in Table S3.

### 2.3. Biomarkers to Distinguish Atopic Subjects

Figure S7 shows how the PCA plot would look like for asthma, non-asthma/non-atopic, and non-asthma atopic subjects if we used only the nine aforementioned biomarkers (i.e., peak IDs: **7, 32, 50, 51, 69, 73, 80, 85, 93** in Table 2). The distribution of the atopic subjects was found to be biased on the asthma side, implying that the pattern of these nine biomarkers from the atopic subjects look more like asthma. Given that atopy is a common underlying feature in asthma, this is unsurprising. Thus, using those nine biomarkers alone, it was difficult to distinguish between asthma and atopic, and between non-asthma/non-atopic and non-asthma/atopic subjects. Therefore, new sets of biomarkers may be needed for further classification.

**Figure 3:**
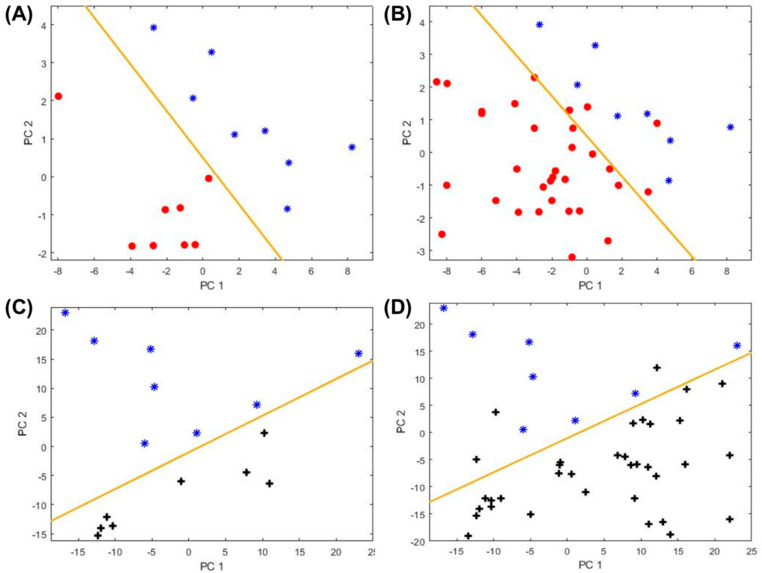
Classification among asthma (red circles), and non-asthma/non-atopic (black crosses), and non-asthma atopic (blue asterisks) subjects. (**A**) PCA plot for the asthma and atopic subjects in the training set using a new set of biomarkers listed in Table 1. (**B**) Corresponding PCA plot for the training set and the testing set combined. (**C**) PCA plot for the non-asthma atopic and non-asthma/non-atopic subjects in the training set using the biomarkers listed in Table 1. (**D**) Corresponding PCA plot for the training set and the testing set combined. The yellow line marks the position of the boundary.

In this study, all of the eight non-asthma/atopic subjects were used in the training set (due to the limited number of subjects), along with another eight randomly selected asthma subjects and eight non-asthma/non-atopic subjects. The remaining asthma subjects and asthma/non-atopic subjects were used as the testing set for validation. The PCA plots are presented in Figure 3, showing significant improvement in distinguishing atopy from asthma and from non-asthma/non-atopic. Two new biomarkers (peak IDs: **6**, **67**) yielded the maximum classification accuracy of 90.5% and the maximum boundary distance for the discrimination between asthma and non-asthma/atopic subjects. Four other biomarkers (peak IDs: **7, 32, 50, 54**) gave the maximum classification accuracy of 93.2% with the maximum boundary distance to distinguish the non-asthma/atopic from the non-asthma/non-atopic group. The corresponding statistics are given in Table S4. Based on the above discussion, atopic subjects can be identified through two steps. First, the nine biomarkers **(7, 32, 50, 51, 69, 73, 80, 85, 93**) are applied to separate out “asthma” and “non-asthma/non-atopic” (note that non-asthma/atopic subjects might be mis-classified as asthma or non-asthma/non-atopic in this step as shown in Figure S7). Then two new sets of biomarkers (**6, 67**) and (**7, 32, 50**, and **54**) are used to further identify atopic from the “asthma” group and “non-asthma/non-atopic” group, respectively.

### 2.4. Biomarkers for Other Asthma Sub-Categories

**Figure 4:**
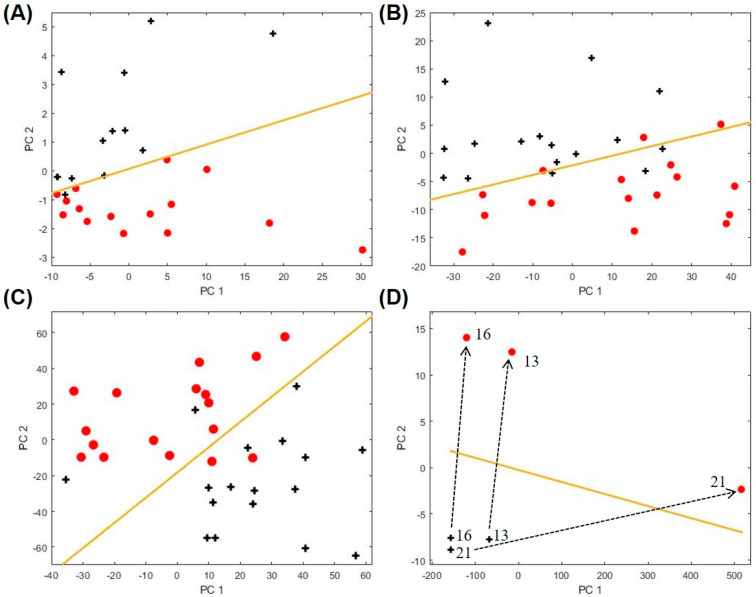
PCA plots for biomarkers of clinically relevant asthma characteristics (**A**) inhaled corticosteroid (ICS) treatment, (**B**) obesity, (**C**) blood eosinophils (EOS) level, and (**D**) upper respiratory illness. The red symbols denote data for the positive groups, i.e., asthma subjects on ICS treatment, obesity (BMI ≥ 30), high EOS level (blood EOS ≥ 0.3), and upper respiratory illness. The black symbols denote the corresponding negative controls, i.e., asthma subjects not on ICS treatment, BMI < 30, low EOS (blood EOS < 0.3), or absent acute upper respiratory illness (baseline sample). The boundary lines are marked in yellow. The corresponding biomarkers are listed in Table 1 and Table 2. The dashed lines show the trajectories in the longitudinal analysis for three asthma patients who later developed upper respiratory illness. The subject IDs are given by the numbers near the crosses. The PCA plot for both training and testing sets for ICS treatment is given in Figure S8.

Using all the subjects in each sub-category and the same procedures described previously, we then identified exhaled biomarkers for the other asthma characteristics of interest, which reflect clinical factors associated with differences in asthma phenotypes and outcomes (ICS treatment, obesity, eosinophil level, and upper respiratory illness; see Table 2). The corresponding PCA plots and the statistics are given in Figure 4 and Table S5, respectively. Particularly, in Figure 4D we show the longitudinal analysis of three asthma patients who later developed upper respiratory illness (URI), demonstrating exhaled breath markers of acute URIs. Overall, the ability to sub-categorize and monitor patients’ trajectories with a non-invasive diagnostic method would provide physicians a tool for streamlined detection and monitoring of asthma phenotypes and outcomes to potentially help define the right treatment protocol.

## 3. Discussion

Non-invasive approaches to understand airway biology in different asthma phenotypes is of great interest, and measurement of VOCs are considered a promising tool [24]. Exhaled breath analysis has advantages over blood and sputum sampling because it is truly non-invasive, easily accessible, low cost, and potentially provides instant results. There is currently no clinically applicable measurement of biomarkers for real-time diagnosis or tracking of asthma phenotypes other than FeNO, which is reflective of eosinophilic airway inflammation and clinical outcomes related to this phenotype. In contrast, non-invasive biomarkers of non-eosinophilic asthma, which encompasses a variety of sub-phenotypes, remain a clinical need. In this pilot study, we expand upon earlier work by showing that exhaled breath analysis can reveal VOCs that discriminate clinically important features of asthma and in the process potentially shed further insight into the airway metabolic basis of such differences. These include distinguishing atopic/non-asthma from asthmatic status, as well as obesity-associated asthma, a phenotype that does not always respond to usual therapies. We also demonstrate VOCs that distinguish asthmatics taking or not taking inhaled corticosteroids. These factors if not considered may impact interpretation of VOC biomarkers and how they inform further study of asthma biology and associated differences in treatment outcomes.

We demonstrate here that a portable GC device can discriminate and identify specific exhaled biomarker compounds that distinguish clinical features of asthma in real-time (which saves time and complexity involved in sample preparation and storage) with high accuracy. Potentially a portable GC analysis system could be developed for home use, which may allow the end user to collect and analyze their breath at home to closely monitor their health condition. To our knowledge, this is the first demonstration of a portable real-time GC system to study breath VOCs in asthma stratified by the additional factors examined, which were chosen for their clinical importance and link to differences in asthma phenotype or outcomes. As previously mentioned these factors might affect interpretation of specific VOCs in asthma. For example, among the nine biomarkers (**7, 32, 50, 51, 69, 73, 80, 85, 93**) delineating asthma from non-asthma/non-atopic subjects, three of them (**7, 32, 50**) overlapped with the biomarkers distinguishing non-asthma/atopic from non-asthma/non-atopic subjects. This highlights that background atopy needs to be considered when interpreting such results, since allergic sensitization is common in asthma. A recent study reported that eNose breathprints could classify atopic and non-atopic subjects with asthma, but specific VOCs contributing to the distinction were not identifiable by this methodology [8]. Notably, we identified two additional compounds, **6** (2-methyl- pentane) and **67** (2,5,9-trimethyldecane), that distinguished asthmatic from non-asthmatic/atopic subjects. Same or similar compounds to these (such as 2-methylpentane, 2,4-dimethylpentane, and branched C_13_ alkanes like 2,3,6-trimethyldecane) were previously reported as markers to distinguish asthma and non-asthma [22,23,25]. Our data suggest that some of these VOCs may further distinguish atopic asthmatic subjects from atopic subjects without asthma.

We identified exhaled biomarkers for other asthma-relevant traits by the same approach [16]. For example, obesity-associated asthma is a significant clinical management problem. Current biomarkers to predict potential response to asthma therapies (e.g., inhaled steroids) do not perform as well in obese patients and correlate poorly with sputum markers of eosinophilic inflammation [26]. In our exploratory study we identified 5 compounds that distinguished obese and non-obese asthmatics. None of these overlapped with biomarker profiles for other group comparisons, except for one compound (**54**, 2,3,6-trimethylheptane) that also distinguished non-asthmatic/atopic from non-asthmatic/non-atopic subjects. The other non-overlapping VOCs identified may be biomarkers of processes that differentiate obese asthma from other phenotypes. Compound **58** (2,6-dimethyl-(*S,E*)-4-octene) in our results is related to 1-octene, a potential marker of oxidative stress, which an earlier study found to be associated with obese status in children compared to lean controls [27]. Breath alkanes are a product of lipid peroxidation [28], and products of lipid peroxidation are known to be associated with airway inflammation and increased asthma severity [29]. We found only one study in the literature that specifically examined breath metabolite profiles in obese and non-obese asthmatics [30]. However, this study used exhaled breath condensate and NMR-based metabolomics, and thus the specific metabolite differences identified in that study cannot be directly compared to the VOCs we identified as distinguishing obese and non-obese asthmatics.

Distinguishing eosinophilic and non-eosinophilic asthma is important because of the therapeutic implications. Eosinophilic asthma, defined by blood or sputum eosinophil numbers, is more responsive to ICS treatment, and as such inhaled steroids are more likely to be prescribed to such patients. This may confound analysis and interpretation of VOCs related to eosinophilic or non-eosinophilic asthma. Moreover, sputum-based determination of eosinophilic asthma is difficult and remains largely a research method. Blood eosinophil counts are often used as a surrogate clinically, but few if any studies have examined breath VOCs between asthmatics categorized by clinically used definitions of high or low blood eosinophils. One study reported 2,6,10-trimethyldodecane as an exhaled biomarker for eosinophilic airway inflammation, which was defined by sputum eosinophil measurements [16]. For comparison, we identified a similar compound, 1-fluorododecane, as one of the biomarkers distinguishing our asthmatics with high or low blood eosinophils counts. A more recent study also used sputum to categorize asthmatics with or without eosinophilic inflammation for breath VOC comparisons [11]. None of the four VOCs we identified as distinguishing asthmatics by blood eosinophil counts overlapped with the findings of Ibrahim et al. [16] and Schleich et al. [11] who used sputum-based stratification. It is known that sputum and blood eosinophil counts are only modestly correlated in asthmatics, and thus the differences between our findings and others reflect different definitions of eosinophilic and non-eosinophilic asthma. Our findings also suggest that the VOCs that we identified may reflect additional biological processes relevant to the presence or absence of asthma-associated systemic eosinophilia.

Inhaled corticosteroids (ICS) are commonly prescribed for asthma and are considered to be more effective for Type 2-driven (eosinophilic) asthma. Inhaled corticosteroid use could also contribute to increased sputum neutrophils, another inflammatory phenotype [31]. Thus, ICS use may confound interpretation of VOCs in asthma. We identified 4 compounds that delineated asthmatics who were or were not on regular ICS therapy. Three of these were uniquely associated with the biomarker profile for this comparison, while **91** (2,6,6-trimethyldecane) also appeared in the biomarker profile that distinguished high/low blood eosinophils. Whether the other three compounds are related to products found in inhaled corticosteroid preparations or reflect aspects of ICS effects or metabolism in the airways will require further investigation. Schleich et al. [11] examined the influence of ICS treatment on the breath VOCs that distinguished asthma sputum inflammatory profiles in their study. It was noted that using five of the VOCs identified, which included differentiation of neutrophilic asthma from other phenotypes, it was not possible to distinguish ICS-treated from non-ICS-using asthmatics in their cohort. One of these compounds (undecane) was also identified in our study (the related compound undecane 3,6-dimethyl) as part of the unique biomarker profile distinguishing asthmatics on or not on regular ICS therapy. Thus, interpretation of VOCs associated with particular inflammation patterns in asthma may be confounded by concurrent treatments for the disease such as ICS. We also identified cyclopropane and chloroacetic acid as unique biomarkers distinguishing ICS users and non-users in our cohort. These two VOCs may or may not directly relate to asthma airway biology. Nonetheless, we speculate they may serve as potential biomarkers of actual ICS use by patients.

Lastly, we explored whether exhaled breath markers might identify asthmatic subjects experiencing acute upper respiratory illness (URI). Longitudinal analysis of three asthma patients revealed that volatile metabolic changes in exhaled breath distinguished URI state from baseline non-ill state in these patients. Interestingly, the biomarker profile consisted of five compounds that did not appear in the other biomarker profiles for our other group comparisons. Although Larstad et al. [20] found that isoprene may be negatively associated with asthma, others have reported the opposite [17] and we found isoprene to be one of the biomarkers that discriminated active upper respiratory illness in our affected asthma patients. Isoprene has been associated with several disease states, thought to relate to cholesterol biosynthesis pathways and is a byproduct of lipid peroxidation [32], thus potentially indicative of increased inflammation. Other VOCs in this respiratory illness-related profile (e.g., 1-cyclopropaneethanol) could reflect escalation of inhaled bronchodilator treatments and components of such therapies. Further longitudinal analysis in more affected patients is needed to follow up this preliminary observation.

Strengths of our study are the demonstration that a portable GC system can be applied to investigate exhaled VOC patterns in real-time and identify specific compounds associated not just with asthmatic state, but also those associated with clinical variables that reflect differences in asthma biology, treatment approach or outcomes. However, this was a pilot proof-of-concept study with only 73 total participants, and our asthma sub-categories had small, if balanced, numbers for comparisons. The major limitation of the study is lack of independent validation groups. A much larger groups of participants are needed to further validate our methods and preliminary findings. However, our preliminary results are encouraging in its demonstration that somewhat unique breath patterns and specific VOCs may be able to discriminate certain asthma sub-types. Identifying specific compounds, rather than just an overall breathprint, also provides important hypothesis-generating information to inform further mechanistic studies of underlying airway pathobiology. This would include how other products of lipid peroxidation, such as the branched alkanes identified in our study and others, contribute to or reflect pathways of airway inflammation involving membrane lipids in asthma [29]. Another limitation comes from the portable GC itself. In order to maintain portability and rapid analysis time, it uses only a 10-m long column and 10 min of separation time. Consequently, it has lower separation capability than benchtop GC that usually uses a 30 m long column and 30–60 min of separation time. In the future, separation capability can be enhanced by developing 2-dimensional GC. Finally, while our vapor detector (μPID) used in this study is very sensitive already [33], recent improvement shows that its sensitivity can be further increased approximately 10-fold. Implementation of the new version of the vapor detector will certainly help detect those VOCs having extremely low concentrations in breath.

In conclusion, exhaled breath VOC profiling is a clinically accessible tool for asthma diagnosis and phenotype assessment, that in combination with other tools, such as nuclear magnetic resonance spectroscopy of exhaled breath condensate, may offer a more comprehensive breathomics approach to asthma evaluation [34]. We demonstrate that the proposed portable GC system is a viable option for rapid real-time assessment in asthma that could be further scaled to point-of-care devices for breath phenotyping in clinical trials as well as in the outpatient clinic.

## 4. Materials and Methods

### 4.1. Description of the Portable GC Device

**Figure 5:**
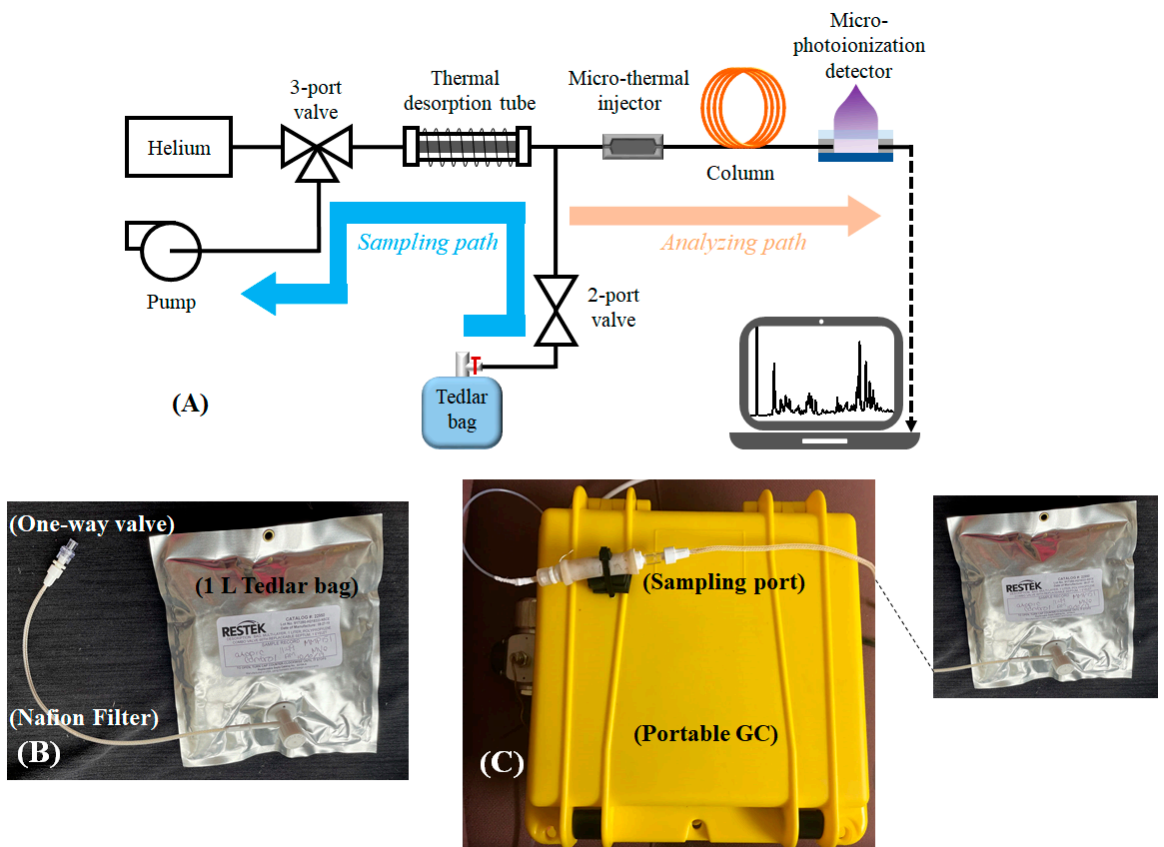
(**A**) Layout of the portable GC device. (**B**) During breath sampling, the subject exhales breath into a Tedlar bag (1 L) via a mouthpiece connected to one-way valve. (**C**) Photo of the portable GC, showing that a Tedlar bag is connected to the sampling port of the device.

The portable GC system used in this study has been reported in our previous work [35,36]. Briefly, as shown in Figure 5A, the GC consists of a thermal desorption tube loaded with Carbopack^TM^ X and B, a micro-thermal injector loaded with Carbopack^TM^ X and B, one 10 m long Agilent J&W DB-1ms, and a micro-photoionization detector. The entire device was housed in a customized plastic case (see Figure 5C) and had a total weight less than 3 kg. LabVIEW^TM^ based codes were developed in-house for the user interface, and device control and automation. The detailed description of the material used, microfabricated components, the preparation of the thermal desorption tube and the column are presented in Sections S1.1 and S1.2 of the Supplementary Materials.

### 4.2. Exhaled Breath Collection and Analysis

Subjects were asked to orally exhale into and fill a 1 L Tedlar bag through a mouthpiece connected to a one-way valve and a Nafion filter in series, as shown in Figure 5B. The one-way valve stops the flow back to the patient mouth, and the Nafion filter is to absorb the moisture content in the breath. The process usually takes about a few minutes. The breath analysis took place either in-situ immediately after the breath sample collection or within 24 h of breath collection. The Tedlar bags were stored under ambient condition until analyzed. During the breath analysis, the Tedlar bag was connected to the sampling port of the portable GC (Figure 5C). The total assay time was 30 min, including 5 min of breath sampling time from the Tedlar bag at a flow rate of 70 mL/min (see the blue path in Figure 5A), 5 min of desorption/transfer time, 10 min of chromatographic separation time (see the orange path in Figure 5A), and 10 min of GC system cleaning time. The detail about the GC system operation is presented in Section S1.3 and also reported in our previous work [35,36].

### 4.3. Chromatogram Processing and Statistical Analysis

Chromatogram preprocessing is critical prior to actual breath analysis. In this work, baseline correction, noise reduction, normalization, peak detection, peak area extraction, and chromatogram aligning is performed prior to the subsequent machine learning and statistical analysis. More detailed description for each step is presented in Section S2 in the Supplementary Materials.

Through machine learning, a subset of peaks (VOCs) were selected as the biomarkers to discriminate between asthma and non-asthma/non-atopic, and among various asthma subcategories based on clinically important asthma characteristics. The statistical analysis method is adapted from our previously published approach [35] based on linear discriminant analysis (LDA) and principal component analysis (PCA) with significant improvement in computation efficiency (Section S3.3.2). The detailed description is elaborated in Section S3 in the Supplementary Materials.

### 4.4. Identification of VOCs Using Mass Spectrometry

The outlet of the portable GC device (i.e., the outlet of the photoionization detector) was coupled to a Thermo Scientific Single Quadrupole Mass Spectrometer (ISQTM Series) for chemical identification of the VOCs in the breath. C_13_ was used as a standard sample for MS calibration. The NIST 2014 library was used for the identification of breath compounds. The results were analyzed with Chromeleon^TM^ 7 Software provided by Thermo Fisher Scientific, Waltha, MA, USA.

## Figures and Tables

**Table 1:** Summary of recruited subjects, the total number of breath samples collected and number available for each clinical characteristic analyzed. ^#^ post-albuterol, mean ± standard deviation. FEV1, forced expiratory volume in 1 s % predicted; ICS, inhaled corticosteroid; BMI, body-mass index.

Category	Non-Asthma, Non-Atopic	Non-Asthma, Atopic			Asthma								
			Total	FEV_1_/FVC ^#^	FEV_1_(% pred) ^#^	ICS Treatment	No ICS Treatment	Obese (BMI ≥ 30)	Non-Obese (BMI < 30)	Blood Eosinophils ≥ 0.3 × 10^9^/L	Blood Eosinophils < 0.3 × 10^9^/L	Upper Respiratory Illness	No Upper Respiratory Illness
Number of subjects	35	8	30	0.78 ± 0.12	97.7 ± 18.7	20	13	17	17	17	17	3	Same 3 subjects as those with upper respiratory illness
Number of breath samples analyzed	37	8	34			21	13	17	17	17	17	3	3
Age, yrs (range)	40.9 (23–71)	29.4 (19–43)	40.2 (18–72)										
Sex, % female	75	50	69										

**Table 2:** Summary of the different study groups and respective identified biomarker IDs.

Study Group		Biomarker Peak ID
Asthma vs. non-asthma/atopic		**6, 67**
Non-asthma/non-atopic vs. non-asthma/atopic		**7, 32, 50, 54**
Asthma clinical variables	Inhaled corticosteroid treatment	**59, 62, 86, 91**
	Obesity	**8, 28, 37, 54, 58**
	Blood eosinophils level	**34, 51, 60, 91**
	Upper respiratory illness	**1, 2, 4, 5, 8**

**Table 3:** Summary of the chemical names, IDs, and retention time of all the biomarkers.

Peak ID	Compound Name	Peak ID	Compound Name
1	2-Methylbutane,	**54**	2,3,6-Trimethylheptane
2	Isoprene	**58**	2,6-Dimethyl (*S*,*E*)-4-octene,
4	1-Cyclopropaneethanol	**59**	1-Butyl-1-methyl-2-propyl- cyclopropane
5	4-Methyl-1-pentene	**60**	1-Fluorododecane
6	2-Methylpentane	**62**	Chloroacetic acid dodecyl ester
7	2-methyl-1-pentene	**67**	2,5,9-Trimethyldecane
8	*n*-Hexane	**69**	2,3,5-Trimethylheptane
28	2,3,4-Trimethylpentane	**73**	2,4,6-Trimethyldecane
32	2,4-Dimethylheptane	**80**	2,6,10,14-Tetramethylheptadecane
34	2-Octene	**85**	2,8-Dimethylundecane
37	2-Methyloctane	**86**	3,6-Dimethylundecane
50	2,2,4-Trimethylheptane	**91**	2,6,6-Trimethyldecane
51	3,3-Dimethyloctane	**93**	5-Methyl-5-propylnonane

## Data Availability

The data are available on request from the corresponding authors due to ongoing study.

## Supplementary Materials

The Supplementary Materials are available online at https://www.mdpi.com/article/10.3390/metabo11050265/s1. Section S1: Portable GC Description and Operation, Section S2: Chromatogram Preprocessing, Section S3: Statistical Analysis for Biomarker Discovery.
