# Supplementary material for: Real Time Breath Analysis Using Portable Gas Chromatography for Adult Asthma Phenotypes
Source: Metabolites. 2021 Apr 23;11(5):265. doi: 10.3390/metabo11050265 (PMC8145057; doi:10.3390/metabo11050265)
Supplement: Supplementary file 1 [file metabolites-11-00265-s001.zip › metabolites-1162653-SI.pdf]

Supplementary Materials for

Real Time Breath Analysis Using Portable Gas Chromatography for Adult Asthma Phenotypes

Ruchi Sharma[1,*], Wenzhe Zang[1,*], Menglian Zhou[1], Lesa Begley[2],
Yvonne Jean Huang[2,+], and Xudong Fan[1,+]

[1] Department of Biomedical Engineering,
University of Michigan, Ann Arbor, MI, U.S.A.

[2]Division of Pulmonary and Critical Care Medicine, Department of Medicine,
University of Michigan, Ann Arbor, MI, U.S.A.

* Equal contribution

+ Corresponding authors: xsfan@umich.edu; yvjhuang@med.umich.edu

Section S1. Portable GC Description and Operation

S1.1. Materials

The DB-1ms Agilent J&W, nonpolar column (length 10 m, i.d. 250 μm, film thickness 0.25 μm) was purchased form Agilent Technologies (P/N: 122-0162, Agilent Technologies). Copper tube (length 10 cm, i.d. 1 mm, o.d. 1.5 mm) was purchased from Swagelok and glass wool was purchased from Sigma Aldrich. Teflon tape was purchased from Grainger (Ann Arbor, MI). Shrink tube was purchased form Digi-Key Electronics. Disposable helium cartridge (95 mL, 2500 psi) was purchased from Leland (South Plainfield, NJ). GC guard columns (i.d. 250 μm and o.d. 380 μm), universal press-tight glass capillary column connectors, and angled Y-connectors were purchased from Restek (Belafonte, PA). The 2-port and 3-port solenoid valves were purchased from Lee Company (Westbrook, CT). A diaphragm pump was purchased from Gast Manufacturing (Benton Harbor, MI). Nickel wire (0.32 mm diameter, 1.24 Ω/m) was purchased from Lightning Vapes (Bradenton, FL). A type K thermocouple was purchased from Omega Engineering (Stamford, CT). A silicon wafer was purchased from University Wafer (Boston, MA). The UV lamps and amplifiers for PIDs were purchased from Baseline-Mocon (Lyons, CO). A 24 V ac/ dc converter was purchased from TDK-Lambda Americas, Inc. (National City, CA). A 24 V and a 12 V ac/dc converters and axial fans were purchased from Delta Electronics (Taipei, Taiwan). Data acquisition cards (DAQ cards), USB-6212 (16 bits) was purchased from National Instruments (Austin, TX). Customized printed circuit board (PCB) was designed and manufactured by M.A.K.S., Inc. (Troy, MI). All the materials are the same as those described in Ref. [1].

S1.2. Design, fabrication, characterization of components and device assembly

The micro-fabricated preconcentrator (μPI) and micro-photoionization detector (μPID) were two microfabricated components used in the present portable GC device. All of these components were fabricated and characterized in-house. The details of μPI and μPID can be found in Ref. [1].

The thermal desorption tube was made of a 5 cm long copper tube with an inner diameter of 1 mm. Both Carbopack™ X and B granules, 10 mg each, were loaded into the hollow cylindrical copper tube using a diaphragm pump. Glass wool was used to separate the Carbopack™ X and B, as well as to seal the copper tube from both ends. Swagelok fittings were used to connect a stainless steel tube of i.d. 250 μm at both the ends of the copper tube. For temperature ramping, the nickel wire was wrapped around the entire length of the copper tube. The nickel wire was insulated from the copper tube using a Kapton tape. A type K thermocouple was attached to the copper tube using a Kapton tape to monitor the temperature in real time. Finally, the thermal desorption tube was preconditioned at 300 °C for 12 h under helium flow.

The 10 m long DB-1MS column for 1D and the nickel wire were placed in parallel, wrapped in Teflon tape, inserted into a shrink tube, and then coiled into a helix of 10 cm in diameter and 1 cm in height. Details can be found in Ref. [1].

As illustrated in Figure 1, the portable GC consisted of a sampling module and an analyzing module. The sampling module consisted of a sampling tube, a thermal desorption tube loaded with Carbopack™ X and B, valves, and a pump. The analyzing module consisted of a μPI loaded with Carbopack™ X and B, a 10 m long Agilent J&W DB-1ms, and a μPID. The modules and components were connected via tubings, universal connectors, and Y-connectors. The entire device was housed in a customized plastic case and had a total weight less than 3 kg, including the weight of the He gas cartridge (231 g), as shown in Figure 1. LabVIEW™ based codes were

developed in-house for the user interface, and device control and automation.

S1.3. Operation of the portable GC

The operation procedures and parameters of the portable GC are described as follows.

(1) Sampling: Breath VOCs were drawn by the diaphragm pump through the 2-port valve and adsorbed by the thermal desorption tube at a flow rate of 70 mL/min for 5 min from a Tedlar bag with a total volume of 350 mL. The optimization of the flow rate resulted from a balance between reducing sampling time and preventing VOC breakthrough in the thermal desorption tube. The sample volume (350 mL) was optimized to achieve adequate signal-to-noise ratios for most VOC peaks while not saturating the detector.

(2) Desorption and injection: The 2-port valve was closed and helium gas was flowed through the 3-port valve to provide the carrier gas at a flow rate of 2 mL/min. Meanwhile, the thermal desorption tube was heated to 300 °C for 5 min to transfer the trapped analytes onto the micro-thermal injector. Then the micro-thermal injector was heated to 250 °C in 0.3 s and then kept at 250 °C for 5 s for complete thermal desorption and injection of the analytes into the column. The micro-thermal injector heating parameter was optimized to desorb all VOCs and achieve sharp injection peak width (~0.5 s full-width-at-half-maximum).

(3) Separation: The analytes underwent separation through the 10 m long column and were then detected by the μPID. During the separation, the column was kept at 25 °C for 2 min, then first ramped at a rate of 10 °C min^{-1} to 80 °C, next ramped at a rate of 40 °C min^{-1} to 120 °C, and kept at 120 °C for 1 min. The helium flow rate was 2 mL/min for the column. The ramp rate, column temperature, and carrier gas flow rate were optimized to achieve the best separation of breath VOCs with the shortest possible time.

(4) Cleaning: After analysis, the thermal desorption tube was heated to 300 ºC for 5 min followed by heating the micro-thermal injector to 250 °C in 0.3 s and then keeping it at 250 °C for 6 s at a helium flow rate of 25 mL/min. This process was repeated twice in order to completely remove residual analytes (if any) trapped in the thermal desorption tube and the micro-thermal injector.

The total assay time was 30 minutes, which included 5 minutes of sample collection, 5 minutes of desorption/transfer, 10 minutes of separation, and 10 minutes of cleaning.

(5) The device was calibrated monthly using a C_6-C_{12} mixture prepared in lab that covers the most range of the VOCs of interest. Meanwhile, the person who conducted the breath analysis has his/her breath samples tested twice on the day before and after the breath analyses on patients were performed.

Section S2. Chromatogram Preprocessing

Chromatogram preprocessing is critical to VOC analysis, because analyte information is often obscured by irrelevant variations from, for example, noise, detector performance, baseline drifting, and peak retention time drifting. These issues exacerbate with breath analysis due to the presence of a large number of VOCs and the large variations in concentrations of those VOCs among different subjects. In this work, the following preprocessing steps are performed with the as-obtained chromatogram raw data (PID signal vs. retention time) prior to the statistical analysis in Section S3.

(1) Baseline correction and removal

In order to correct the baseline drifting, which typically arises from column stationary phase bleeding and/or detector sensitivity variations, the open-source adaptive iterative reweighted Penalized Least Squares (airPLS) algorithm is adopted[2], which iteratively alters weights of sum squared errors (SSE) between the previously established baseline and the original signals. The baseline noise signal is numerically centered around zero after the baseline correction.

(2) Noise reduction

After the baseline correction, the signal is de-noised via the locally weighted scatterplot smoothing (LOWESS) approach[3]. As a result, the signal-to-noise (S/N) ratio in chromatograms is further improved.

(3) Normalization

The total area under the chromatogram profile is normalized to unity after the baseline removal and noise reduction, as described previously[4]. The sum-normalization approach is commonly used in GC-based breath analysis[5-10].

(4) Peak detection

The chromatogram curve is scanned for local maxima and the associated peak apex positions (*i.e.*, retention times), peak heights, and endpoints[11]. Peaks that do not exceed the cutoff height (*e.g.*, detector detection limit) and/or the pre-defined full width at half maximum (FWHM) will be filtered out.

(5) Peak coelution identification

Peak coelution is generally unavoidable in the chromatogram of exhaled breath, due primarily to the presence of the large number of VOCs. A peak with a coelution issue is identified if the height of either of its two endpoints (left and right) is above the 1/10 of the peak apex height (Figure S1A); otherwise the coelution is deemed negligible. Coelution occurs with at least two adjacent peaks. A group of coeluted peaks is identified (Figure S1B) when the height of the left endpoint of the first peak and the right endpoint of the last peak are close to baseline and all middle end points are above the cutoff criteria (*e.g.*, >1/10 of the peak height).

(6) Peak area extraction

For a fully separated peak (*i.e.*, no coelution issue), its area, which is related to the amount or concentration of the corresponding compound, is extracted by integrating the area between its two endpoints. For the group of multiple coeluted peaks, deconvolution is performed. Briefly, a

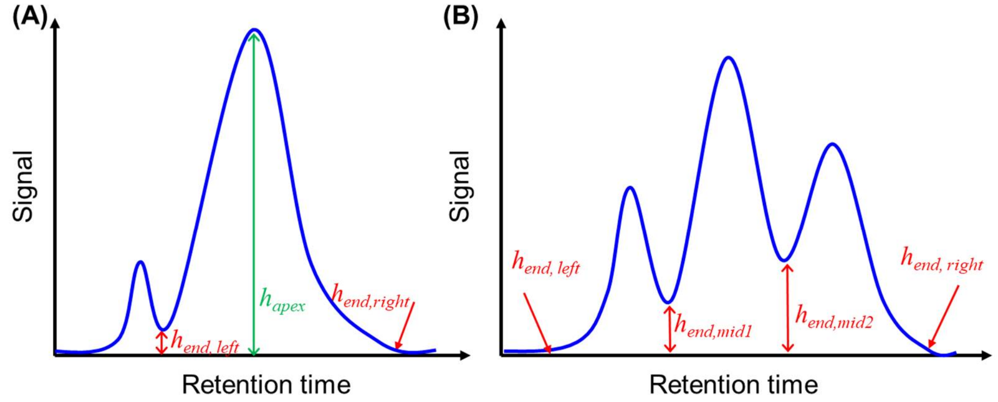

Figure S1. Conceptual illustration of peak coelution identification. **(A)** A peak with a coelution issue is identified if its height ($h_{end,left}$ and $h_{end,left}$, marked in red) of either left or right endpoint is above 1/10 of the peak apex height (h_{apex}, marked in green); otherwise the coelution is deemed negligible. The positions of the two endpoints and apex is extracted in Step 4. **(B)** A group of coeluted peaks are identified if the height at the left endpoint of the first peak ($h_{end,left}$) and the right endpoint of the last peak ($h_{end,left}$) are very close to the baseline, the height at the rest end points in the middle ($h_{end,mid}$) are above the cutoff criteria.

Gaussian function is used as the fitting function and the extracted parameters in Step 4 are used as the initial fitting parameters for each coeluted peak. Then iterative deconvolution of the group of the coeluted peaks is performed using Nelder-Mead Modified Simplex until the fitting error is below a predefined value. The total peak area and peak profile of the coeluted peaks are preserved during the fitting. The integration of each deconvoluted Gaussian curve is extracted as the corresponding peak area.

(7) Retention time alignment

The correlation optimized warping (COW) method is applied to all measured chromatograms to correct the peak retention time drift due to fluctuations in experimental conditions (*e.g.*, flow rate, column temperature programming profile, and ambient temperature). COW is a widely used algorithm for globally optimized local alignment[12,13]. The aligning is achieved by dividing a chromatogram into a number of local segments and each segment is iteratively stretched/compressed by interpolation until correlation between the sample and reference chromatograms is maximized. Note that in this work only the retention time of each peak is altered by the COW aligning, whereas the peak area remains the same. We notice that although after the COW aligning, the peak of the same compound among all the chromatograms may fail to yield the exactly same value. The aligning errors for all peaks are below 0.75 seconds, which are far below a typical distance between two adjacent peaks in the chromatogram (Figure S2). Therefore, the peaks whose retention time are within a ±0.75 seconds window (or slot) defined by the peak in the reference chromatogram are treated as the same peak.

(8) Consolidation

After the retention time alignment, there are a total of 103 peaks found across the chromatograms of all the study subjects in the current work. Each peak may represent only one (no coelution) or multiple VOCs (complete coelution). Note that not all the 103 peaks are present

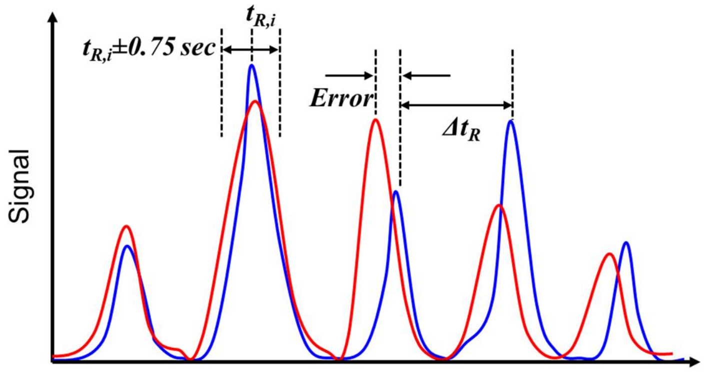

Figure S2. Conceptual illustration of retention time grouping after the COW aligning, where the blue chromatogram represents the reference chromatogram and the red one represents the chromatogram from the sample under test, which has been aligned to the blue reference chromatogram with COW. The aligning errors for the peaks of the same compound across different chromatograms (labelled as "Error" in the figure) are noticed to be below 0.75 seconds, which are much smaller than a typical distance between two adjacent peaks in the same chromatogram (labelled as "Δt_R" for the 3rd and 4th peaks in the blue reference chromatogram). Any peak in the sample chromatogram whose retention time is within a ±0.75 seconds window (or slot) defined by retention time of one peak ($t_{R,i}$, where i represents one particular peak) in the reference chromatogram is treated as the same peak.

in the chromatogram of one particular study subject and the areas of absent peaks are assigned to zero. The normalized peak areas of each chromatogram are sorted based on the aligned retention times ascendingly and all the peaks are assigned with the peak IDs as 1, 2, …, and 103. The normalized area of all the 103 peaks among all the study subjects can be consolidated as a matrix:

$$\begin{pmatrix} x_{1,1} & \cdots & x_{1,n} \\ \vdots & \ddots & \vdots \\ x_{m,1} & \cdots & x_{m,n} \end{pmatrix}, (1)$$

where each element $x_{i,j}$ represents the normalized peak area of the ith patient and the jth peak. m is the total number of the study subjects and all the study subjects are assigned with subject IDs as 1, 2, …, and m. and n is the total number of the peaks, which is 103 in the current work.

Similarly, a classifier matrix can be formed based on the medical adjudication of the study subjects, *i.e.*, 1 for the positive group and 0 for the control (or negative) group:

$$\begin{pmatrix} c_1 \\ \vdots \\ c_m \end{pmatrix}. (2)$$

Section S3 Statistical Analysis for Biomarker Discovery

The statistical analysis method is adapted from our previously published approach[4] based on principal component analysis (PCA) and linear discriminant analysis (LDA) with significant improvement in computation efficiency.

S3.1. Enumerating all possible peak subsets

Assuming that there are a total of N out of the total 103 detected peaks that are relevant to the classification between the positive group and the control group, a total of *C(103,N)* peak combinations can be generated, each of which is a subset of the 103 peaks. *C(103,N)* is a combinatorial number that becomes extremely large when N is above 5. The normalized peak areas of one particular N-peak subset can be expressed as an m by N matrix:

$$\begin{pmatrix} x_{1,k_1} & \cdots & x_{1,k_N} \\ \vdots & \ddots & \vdots \\ x_{m,k_1} & \cdots & x_{m,k_N} \end{pmatrix}, (3)$$

where m is the number of study subjects. Each element $x_{i,j}$ represents the normalized peak area of the ith patient and j^{th} peak, which serves as the feature of linear discriminant analysis (LDA) and principal component analysis (PCA) in subsequent Sections S3.3-3.5. (k_1, k_2, …, k_N) are the peak IDs in one particular N-peak subset.

S3.2. Training set and testing set

The subjects for asthma study are randomly divided into the training set (total 45 study subjects) and the testing set (total 25 study subjects). The training set is used to select the best peak combination (*i.e.*, the set of biomarkers) and determine the associated linear boundary for the best classification result (see Section S3.3 later), whereas the testing set is used for validation (see Section S3.4 later).

For the group of asthma confounding factors (atopic control, obesity, upper respiratory illness, and eosinophils level), all the study subjects are involved into the training set for biomarker discovery due to the limited number of study subjects. For the asthma confounding factor of ICS treatments, all the 13 samples from subjects who receive ICS treatment plus 15 non-ICS samples are involved into the training sets. The rest 6 ICS samples are used for validation.

S3.3. Peak subset selection – biomarker discovery

When N=1, only one peak is used as the biomarker to distinguish the positive group and the control group. The highest binary classification accuracy that can be achieved with only one peak (*i*.e., optimal classification accuracy with one peak) is expected to be low. When N increases (*e.g.*, N=2, 3, …, and 9, *etc.*), the optimal classification accuracy that can be achieved with N peaks increases, as more peaks are added to the biomarker subset (Figure S3). With further increased N, the optimal classification accuracy levels off, as additional peaks do not contribute to the distinction between the positive group and the control group. However, when N continues to increase, the optimal classification accuracy that can be achieved with N peaks starts to deteriorate, as additional peaks impair the classification ability. Eventually, when N=n (n is the total number of the peaks and n=103 in the current work), that is, when all peaks are used as the biomarkers, there is no distinction between the positive and the control groups. Therefore, we

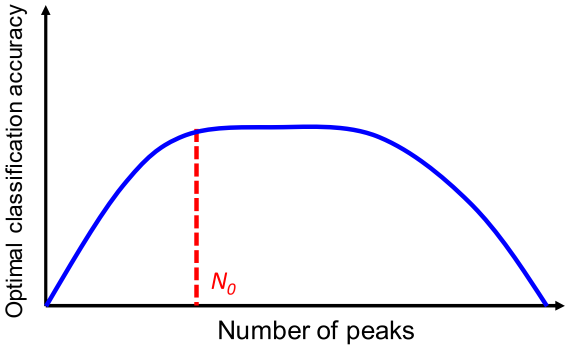

Figure S3. Conceptual illustration of the trend of optimal classification accuracy with the increased number of peaks used in the biomarker set.

expect that the number of peaks that best distinguish between the positive and the control groups should lie between 1 and n, *i.e.*, $1<N<n$. Note that the same optimal classification accuracy can possibly be achieved with different number of peaks, for example, N_0, N_0+1, and N_0+2 peaks (see Figure S3 for illustration). If this is the case, we choose the subset that has the minimal number of peaks (*i.e.*, N_0) as the biomarker set. N_0 is the minimum number of required biomarkers to achieve the highest classification accuracy.

There are two different yet equivalent approaches to select the biomarker set.

S3.3.1. Independent peak subset selection (first approach)

The first approach is straightforward, but becomes time consuming with increased N. In this approach, the LDA-AUC model is performed with all possible N-peak subsets. Briefly, for each subset of peaks, the corresponding normalized peak area matrix in Eq. (3) and the classifier matrix in Eq. (2) are coupled to train an LDA model and its area under the corresponding receiver operating characteristic curve (AUC, ROC) is calculated. The subset with the highest AUC value gives the optimal classification result. As illustrated in Figure S3, when N increases, the optimal classification accuracy increases and then becomes leveled off. For example, if the optimal N_0-peak subset provides the same accuracy as the optimal (N_0+1)-peak subset, the peak search can stop and the optimal N_0-peak subset is fixed as the biomarker set that contains the selected N_0 peaks. Next, PCA analysis is performed with the normalized peak area of the selected biomarkers as the features, and the principal component (PC) coefficients and the linear classification boundary line are extracted, which will be validated in the testing set in Section S3.4.

The number of subsets that contain N peaks is given by $C(103,N)$, which is a combinatorial number and can be extremely large when $5<N<98$. Therefore, when N is above 5, the LDA-AUC calculation for all $C(103,N)$ subsets becomes prohibitively time consuming. In this work, the independent peak subset selection approach is applied to N=1, 2, …, 5, the top fifteen peak subsets of which are listed in Table S1. The PC scores of the training set for asthma analysis are plotted in Figure S4 using the optimal peak subsets listed in Table S1.

S3.3.2. Iterative peak subset selection (second approach)

When N is above 5, the second approach is used that significantly reduces the computational burden. First, the independent peak subset selection approach (*i.e.*, the first approach) is applied to all the peak subsets that contain 2-5 peaks. Since the total number of the subsets for $N<6$ is relatively small, the computation time is affordable. If the classification accuracy levels off or starts to deteriorate, for example, at the 5-peak subsets, *i.e.*, the classification accuracy generated

No.	2-peak subsets	AUC	ID 1	ID 2	3-peak subsets	AUC	ID 1	ID 2	ID 3
1		0.889	45	50		0.950	45	47	50
2		0.870	14	80		0.937	14	46	73
3		0.866	14	73		0.928	14	73	80
4		0.866	26	80		0.924	26	80	93
5		0.866	47	50		0.911	14	73	89
6		0.865	14	21		0.908	47	50	89
7		0.864	45	54		0.908	45	50	52
8		0.863	48	70		0.908	45	50	92
9		0.858	14	54		0.907	14	73	85
10		0.858	25	80		0.907	14	45	50
11		0.858	44	45		0.907	46	50	51
12		0.858	45	51		0.907	50	51	80
13		0.858	50	77		0.907	50	79	83
14		0.857	50	81		0.905	14	43	73
15		0.854	21	50		0.905	14	73	91

No.	4-peak subsets	AUC	ID 1	ID 2	ID 3	ID 4	5-peak subsets	AUC	ID 1	ID 2	ID 3	ID 4	ID 5
1		0.973	14	46	64	73		0.987	32	50	51	80	93
2		0.971	45	47	50	95		0.986	25	38	60	62	79
3		0.965	25	35	64	80		0.986	45	47	50	61	95
4		0.965	45	47	50	61		0.984	25	35	52	64	80
5		0.963	45	47	50	52		0.981	14	25	35	64	80
6		0.961	40	45	47	50		0.981	14	46	56	64	73
7		0.961	45	47	50	55		0.979	14	45	47	50	95
8		0.961	45	47	50	72		0.979	14	46	58	64	73
9		0.960	45	46	47	50		0.979	25	35	64	80	83
10		0.958	20	45	47	50		0.978	14	46	64	73	91
11		0.958	44	45	47	50		0.978	14	46	64	73	94
12		0.958	45	47	48	50		0.978	14	46	64	73	95
13		0.958	45	47	49	50		0.978	20	25	35	64	80
14		0.958	14	45	47	50		0.978	24	45	47	50	95
15		0.958	45	47	50	70		0.978	45	47	50	53	95

Table S1. 2-, 3-, 4-, and 5-peak subsets with the top fifteen AUC values for the classification of asthma and non-asthma subjects (obtained from the first approach). The optimal peak subset is shown in red.

by the optimal 4-peak subset is the same as or better that of any 5-peak subset, then this optimal 4-peak subset is selected as the biomarker set for classification. If the optimal 5-peak subset has a higher classification accuracy than any 4-peak subset, then we proceed to 6-peak subsets. Instead of enumerating all *C(103,6)* (~1.43E9) 6-peak subsets and conducting independent peak search (*i.e.*, the first approach described in Section S3.3.1), we form 6-peak subsets by respectively pairing a selected number of the top 5-peak subsets with one additional peak, the total number of which is much lower than *C(103,6)*. For example, we can select the top 2000 5-peak subsets that have the highest AUC values, each of which is respectively paired with one additional peak from the remaining 103-5=98 peaks. A total number of 2000 × (103-5) = 196,000 (about 7,300 times smaller than *C(103,6)*) 6-peak subsets can be formed to train the LDA-AUC model. Note that there are multiple duplicated subsets (*i.e.*, all peak IDs are same) among the above 196,000 paired 6-peak subsets, which need to be removed prior to the training the LDA-

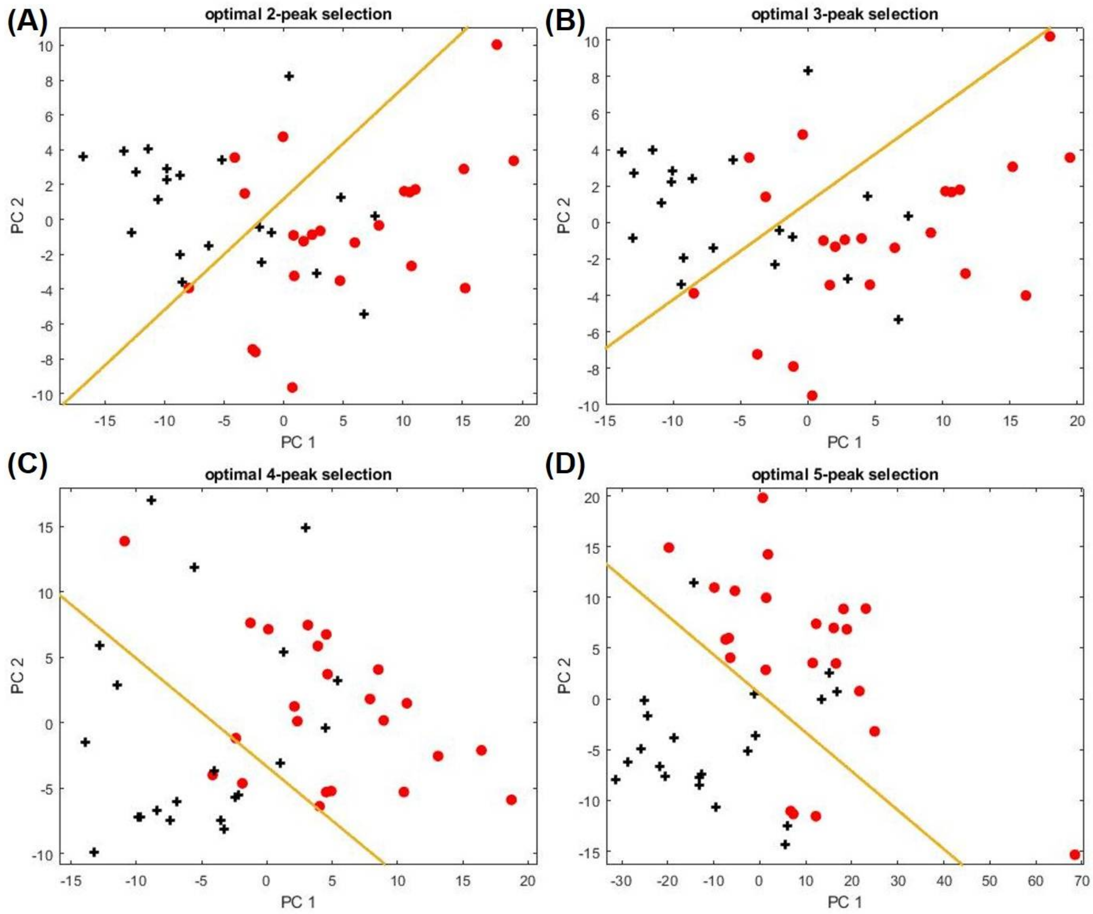

Figure S4. PCA plots of the asthma training set using the optimal 2-, 3-, 4-, and 5-peak subsets listed in Table S1, which show progressively improved classification accuracy (75.6%, 77.8%, 80.0%, and 84.4% from A to D) with increased peak number. The red and black symbols denote respectively the asthma and non-asthma subjects. The yellow line marks the position of the boundary. Note that the atopic subjects are excluded from both asthma and non-asthma groups.

AUC models. Therefore, the total number of the final 6-peak subsets for the model training is much smaller than that generated by the independent peak selection in the first approach, which significantly saves computation time. Similarly, if the optimal 6-peak subset has a higher classification accuracy than any 5-peak subset, then we proceed to 7-peak subsets. The above process continues until the classification accuracy levels off or starts to deteriorate. The top 15 6-peak combinations with the highest AUC values for asthma study is given in Table S2 with the AUC of the optimal subset reaching one.

To be more general, once we have completed the *N*-peak subset selection, instead of conducting independent (*N*+*N'*)-peak search described in the first approach, we can employ an iterative way (Figure S5) based on the top *p* sets of *N*-peak combinations that are obtained from the independent *N*-peak selection approach (*i.e.*, the first approach). Each of the best *p* sets of *N*-peak combinations is paired with all possible *N'*-peak subsets formed from the remaining (*n*-*N*) peaks. Therefore, a total of $p \times C(n\text{-}N, N')$ (*N*+*N'*)-peak subsets can be formed. One such subset is exemplified as follows.

No.	AUC	ID 1	ID 2	ID 3	ID 4	ID 5	ID 6
1	1	14	32	50	51	80	93
2	0.9984	32	50	51	52	80	93
3	0.9968	14	29	34	54	56	71
4	0.9968	25	38	64	80	81	86
5	0.9968	25	38	64	80	86	96
6	0.9952	7	32	50	51	80	93
7	0.9952	14	25	38	60	62	79
8	0.9952	14	29	34	54	56	64
9	0.9952	25	50	51	64	80	81
10	0.9952	25	50	51	64	80	96
11	0.9952	32	50	51	78	80	93
12	0.9936	1	32	50	51	80	93
13	0.9936	14	25	37	38	60	62
14	0.9936	25	38	60	62	71	79
15	0.9936	32	33	50	51	80	93

Table S2. 6-peak subsets with the top fifteen AUC values for the classification of asthma and non-asthma subjects (obtained from the second approach). The optimal peak subset is shown in red.

$$\begin{pmatrix} x_{1,k_1} & \cdots & x_{1,k_N} & x_{1,k_1'} & \cdots & x_{1,k_{N'}'} \\ \vdots & \ddots & \vdots & \vdots & \ddots & \vdots \\ x_{m,k_1} & \cdots & x_{m,k_N} & x_{m,k_1'} & \cdots & x_{m,k_{N'}'} \end{pmatrix}, (4)$$

where m is the total number of the study subjects, (k_1, k_2, …, k_N) refers to one of the best p sets of N-peak combinations. (k_1', k_2', …, $k_{N'}'$) is one of the N'-peak combinations selected from the remaining (n-N) peaks. N' is usually 1 or 2, meaning that each time we add only 1 or 2 new peaks to the N-peak subsets.

Next, the LDA-AUC model is trained for all the above (N+N')-peak subsets with duplicates removed, and all the combinations are sorted descendingly based on the AUC values. However, some peaks in the independent (N+N')-peak selection may not appear in the top p sets of the N-peak combinations. To circumvent this issue, we add additional top q sets of N-peak (for a total of top (p+q) N-peak combinations), and iteratively conduct another round of the (N+N')-peak selection until the top l (N+N')-peak subsets reach convergence (*i.e.*, the peak IDs in each of the top l (N+N')-peak subsets are exactly the same between the adjacent two rounds of peak subset selection). In this work, the second approach is adopted when N is above 5 (N=6, 7, 8, and 9, *etc.*) and typical values of p, q, and l used in this work are 2000, 1000, and 20000, respectively.

Below we explain in detail why we need to choose multiple top N-peak subsets rather than only the optimal N-peak subset (only the top 1) during the creation of (N+N')-peak subsets and validate the equivalence of the above two peak subset selection approaches by showing that they produce the same optimal peak subset.

Let us first take a look at the 3-, 4-, and 5-peak selection results in Table S1 using the data from the asthma breath analysis study. There are no identical peaks between the optimal 3-peak subset and the optimal 4-peak subset (Note that the optimal peak subset has the highest AUC value among all peak subsets that have the same number of peaks in the subset). In fact, three of the peaks (peak ID = 14, 46, and 73) in the optimal 4-peak subset first appear in the 2nd best 3-peak subset and the last one (peak ID = 64) does not emerge until the 44th best 3-peak subset.

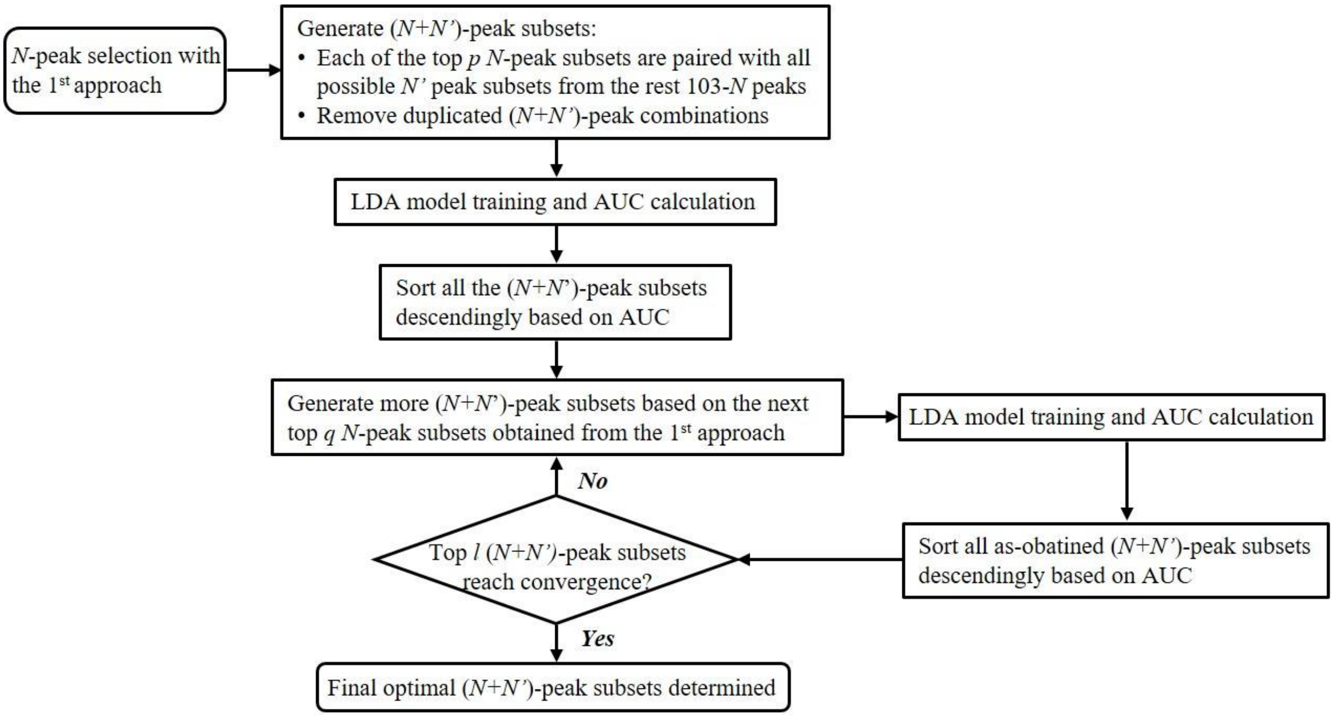

Figure S5. Flow chart of the iterative peak subset selection (second approach).

The physical interpretation is that although any 3-peak subsets formed by three of the four peaks in the optimal 4-peak subset (peak ID = 14, 46, 64, and 73) is inferior to the optimal 3-peak subset (peak ID = 45, 47, and 50), the four peaks (peak ID = 14, 46, 64, and 73) collectively yield an AUC higher than that for the optimal 3-peak subset. Similarly, the optimal 4-peak subset (peak ID = 14, 46, 64, and 73) with AUC = 0.973 and the optimal 5-peak subset (peak ID = 32, 50, 51, 80, and 93) with AUC = 0.987 have no peaks in common. Therefore, in the iterative peak subset selection approach, we cannot choose only the optimal N-peak subset (*i.e.*, top 1 N-peak subset) in order to create (N+1)-peak subsets (by adding an additional peak to the optimal N-peak subset) or (N+N')-peak subsets (by additional N' peaks to the optimal N-peak subset). For example, we would never find the optimal 5-peak subset (peak ID = 32, 50, 51, 80, and 93), if we start with only the optimal 4-peak subset (peak ID = 14, 46, 64, and 73). Interestingly, peak 32, 50, 51, 80, and 93 appear in the 22nd, 2nd, 51st, 35th, and 50th best 4-peak subset. Therefore, when we create the 5-peak subsets, we should not only use the best (top 1) 4-peak subset with the highest AUC value, but also include multiple top 4-peak subsets with top AUC values (*e.g.*, 2nd, 3rd, …., 1000th). After we train the LDA-AUC model on those newly created 5-peak subsets, we can reproduce all of the top (*e.g.*, top 20000) 5-peak subsets that are identical to those obtained from the independent 5-peak subset selection approach (*i.e.*, the first approach). Note that here we use 3-, 4-, and 5-peak subsets for illustration purposes. The same phenomenon holds for the subsets with higher number of peaks. In practice, we enumerate all 2-, 3-, 4-, and 5-peak subsets since the total number of these subsets is relatively low and the computation time is short to calculate the AUC values for all of them. The iterative peak subset selection approach (*i.e.*,

the second approach) starts from 6-peak subsets by adding one new peak to the top (top 2000 in this work) 5-peak subsets.

To validate the equivalency between the first and the second approach, both the two approaches are used in 3-peak, 4-peak and 5-peak subset selection for the asthma study. First, independent peak search (*i.e.*, first approach) is applied respectively to 2-peak, 3-peak, 4-peak and 5-peak selections. Next, we start from the top 1000 2-peak subsets and top 2000 3-peak subsets, and add one or two peaks to perform the iterative peak selections (*i.e.*, (2+1)-, (2+2), (3+1)-, (3+1+1)- and (3+2)-peak selections). All of the top 20000 3-peak, 4-peak, and 5-peak subsets match between the two approaches.

S.3.3.3. Additional selection criterion

We notice that when N increases, the AUC values approaches 1. For example, when N=6 in our asthma study, the AUC value for the optimal 6-peak subset (see Table S2) becomes 1, and when N=7 multiple 7-peak subsets have the AUC of 1 (so do many 8- and 9-peak subsets), meaning that all of them are the optimal peak subset based on the AUC value. In order to further select the optimal peak subset for best classification, we add an additional selection criterion. PCA analysis of each peak subset with AUC=1 is conducted with the training set, which yields the PC coefficients and a linear boundary line between the positive group and the control (or negative) group. All the peak subsets with AUC=1 are further sorted descendingly based on the Fisher criterion function

$$J = \frac{d^2}{s_{positive}^2 + s_{ctrl}^2}, \quad (5)$$

where d is the distance between the mean values of the positive group and the control groups. $s_{positive}$ and s_{ctrl} are the scatter of the data points of each group. The physical interpretation of Eq. (5) is that the distance between the means should be as large as possible and the data variation around the mean within each category needs to be minimized. The biomarker set is the one that has the largest J.

S.3.3.4. Biomarkers to distinguish asthma and non-asthma

Figure S4 shows the PCA plots for 2-, 3-, 4-, and 5-peak subsets for the training set (45 subjects). As discussed previously, the classification accuracy is improved progressively from 75.6% to 84.4%. The classification accuracy is further improved when more peaks are added (see Figure S6) until 9 peaks (97.8%). The optimal 10-peak subset yields the same classification performance as the optimal 9-peak subset. Therefore, we stop at the 9-peak subsets. Then we apply the additional selection criterion (Eq. (5)) to those 9-peak subsets that have the same highest classification accuracy and obtain the biomarker set shown in Table 3. The corresponding statistics is summarized in Table 4.

S3.4. Testing set validation

With the PC coefficients acquired from the training set in Section S3.3., the PC scores can be calculated for the testing set (25 subjects) and shown in Figure 2. The corresponding statistics is given in Table 2.

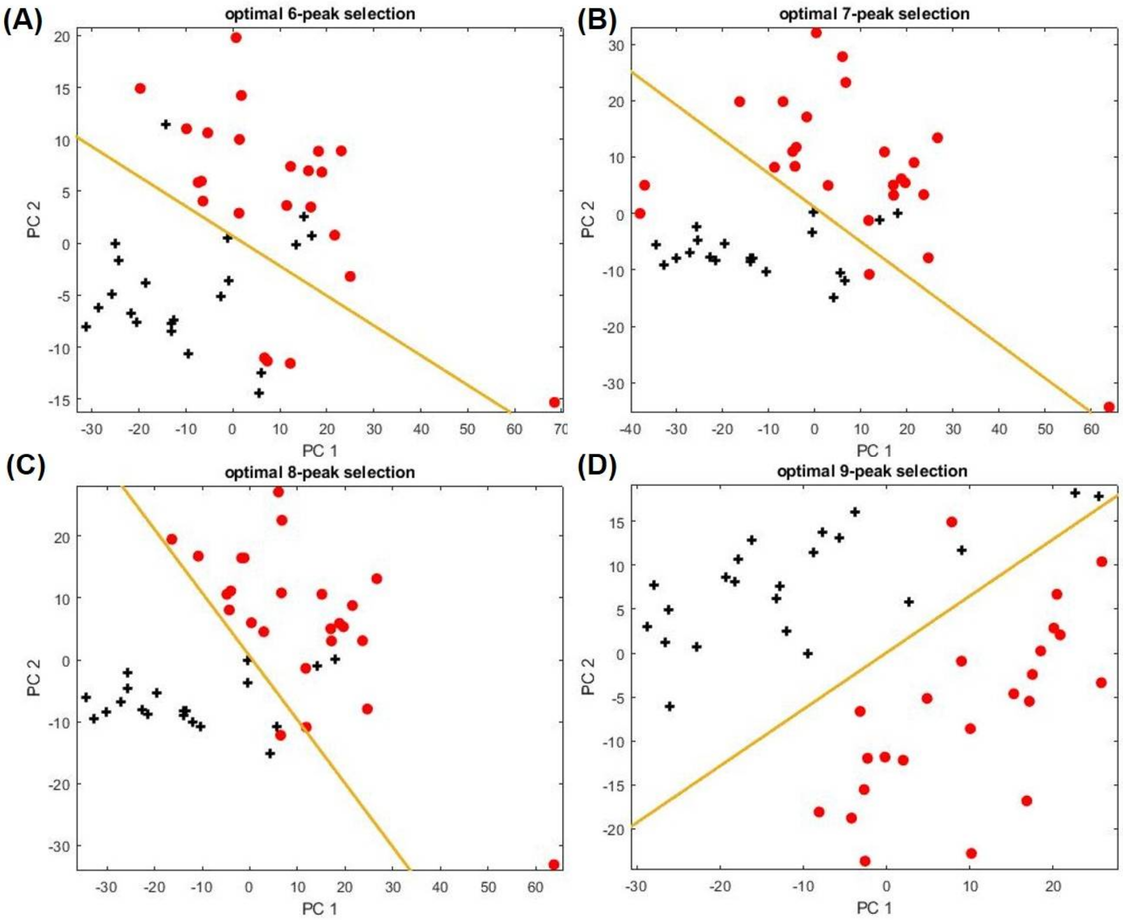

Figure S6. PCA plots of the asthma training set using the optimal 6-, 7-, 8-, and 9-peak subsets, which show progressively improved classification accuracy (84.4%, 88.9%, 93.3%, and 97.8% from A to D) with the increased number of biomarkers. The optimal peak subsets for A-D are (14, 32, 50, 51, 80, 93), (18, 32, 50, 51, 52, 80, 93), (16, 32, 50, 51, 52, 75, 80, 93), and (7, 32, 50, 51, 69, 73, 80, 85, 93), respectively. The red and black symbols denote respectively the asthma and non-asthma subjects. The yellow line marks the position of the boundary. Note that the atopic subjects are excluded from both the asthma and non-asthma groups.

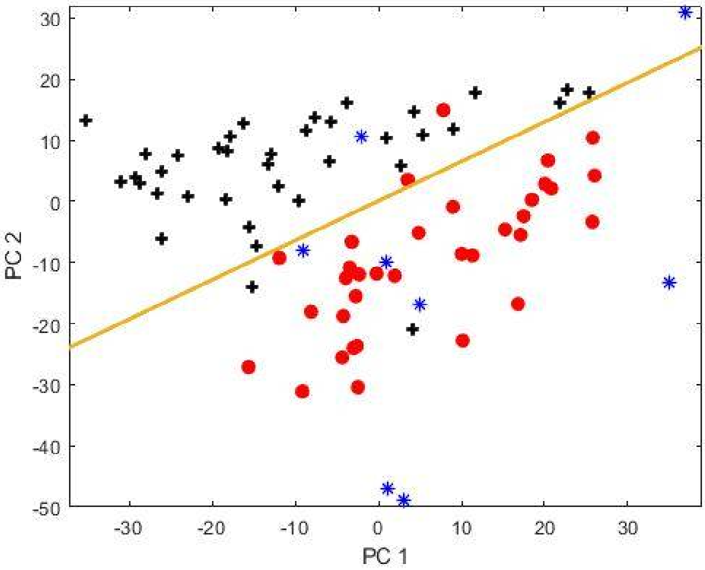

Figure S7. Classification among asthma (red circles), and non-asthma/non-atopic (black crosses), and non-asthma atopic (blue asterisks) subjects using the PC scores for the atopic subjects obtained using the 9 biomarkers listed in Table 2. The distribution of the atopic subjects is found to be biased on the asthma side.

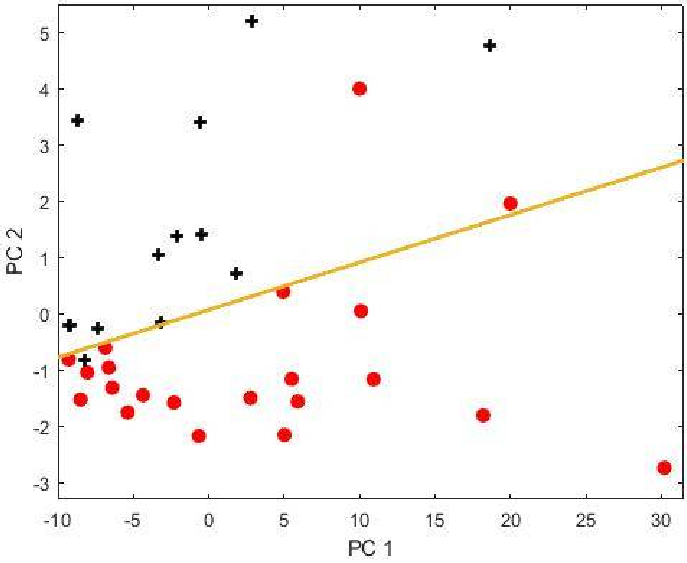

Figure S8. PCA plot of both training sets and testing sets (additional 6 ICS samples) using the optimal biomarkers in Table 2.

Section S3 Statistics Summary

Training set			
	**Asthma**	**Non-asthma**	**Row total**
**Positive**	22	0	22
**Negative**	1	22	23
**Column total**	23	22	45
**Specificity**	100%		
**Sensitivity**	95.7%		
**Positive predictive value**	100%		
**Negative predictive value**	95.6%		
**Total accuracy**	97.8%		
Testing set			
**Positive**	10	2	12
**Negative**	1	13	13
**Column total**	11	15	26
**Specificity**	86.7%		
**Sensitivity**	90.9%		
**Positive predictive value**	83.3%		
**Negative predictive value**	92.9%		
**Total accuracy**	88.5%		
Training + Testing set			
**Positive**	32	2	34
**Negative**	2	35	37
**Column total**	34	37	71
**Specificity**	94.6%		
**Sensitivity**	94.1%		
**Positive predictive value**	94.1%		
**Negative predictive value**	94.6%		
**Total accuracy**	94.4%		

Table S3. Statistics summary for asthma vs. non-asthma/non-atopic.

Asthma vs. Atopic

Training set			
	**Asthma**	**Non-asthma/atopic**	**Total**
**Positive**	8	0	8
**Negative**	0	8	8
**Column total**	8	8	16
**Specificity**	100%		
**Sensitivity**	100%		
**Positive predictive value**	100%		
**Negative predictive value**	100%		
**Total accuracy**	100%		

Training set + Testing set			
	**Asthma**	**Non-asthma/atopic**	**Total**
**Positive**	30	0	30
**Negative**	4	8	12
**Column total**	34	13	42
**Specificity**	100%		
**Sensitivity**	88.2%		
**Positive predictive value**	100%		
**Negative predictive value**	66.7%		
**Total accuracy**	90.5%		

Atopic vs. Non-Asthma and non-Atopic

Testing set			
	**Non-asthma/atopic**	**Non-asthma/non-atopic**	**Total**
**Positive**	8	0	8
**Negative**	0	8	8
**Column total**	8	8	16
**Specificity**	100%		
**Sensitivity**	100%		
**Positive predictive value**	100%		
**Negative predictive value**	100%		
**Total accuracy**	100%		

Training set + Testing set			
	**Non-asthma/atopic**	**Non-asthma/non-atopic**	**Total**
**Positive**	8	3	11
**Negative**	0	33	33
**Column total**	8	36	44
**Specificity**	91.7%		
**Sensitivity**	100%		
**Positive predictive value**	72.7%		
**Negative predictive value**	100%		
**Total accuracy**	93.2%		

Table S4. Statistics summary for asthma, atopic control, and non-asthma/non-atopic.

ICS treatment				
**Training set**		**ICS**	**Non-ICS**	**Total**
	**Positive**	15	1	16
	**Negative**	0	12	12
	**Column total**	15	13	28
	**Specificity**	92.3%		
	**Sensitivity**	100%		
	**Positive predictive value**	93.8%		
	**Negative predictive value**	100%		
	**Total accuracy**	96.4%		
**Training set + testing set**	**Positive**	20	1	21
	**Negative**	1	12	13
	**Column total**	21	13	34
	**Specificity**	92.3%		
	**Sensitivity**	95.2%		
	**Positive predictive value**	95.2%		
	**Negative predictive value**	92.3%		
	**Total accuracy**	94.1%		

Obesity			
	**BMI ≥ 30**	**BMI < 30**	**Total**
**Positive**	14	3	17
**Negative**	3	14	17
**Column total**	17	17	34
**Specificity**	82.4%		
**Sensitivity**	82.4%		
**Positive predictive value**	82.4%		
**Negative predictive value**	82.4%		
**Total accuracy**	82.4%		

EOS level			
	**EOS ≥ 0.3**	**EOS < 0.3**	**Total**
**Positive**	15	2	17
**Negative**	2	15	17
**Column total**	17	17	34
**Specificity**	88.2%		
**Sensitivity**	88.2%		
**Positive predictive value**	88.2%		
**Negative predictive value**	88.2%		
**Total accuracy**	88.2%		

Upper respiratory illness

	Positive	Negative	Total
**Positive**	3	0	3
**Negative**	0	3	3
**Column total**	3	3	6
**Specificity**	100%		
**Sensitivity**	100%		
**Positive predictive value**	100%		
**Negative predictive value**	100%		
**Total accuracy**	100%		

Table S5. Statistics summary for the other clinical variables.

References

(1) Lee, J.; Zhou, M.; Zhu, H.; Nidetz, R.; Kurabayashi, K.; Fan, X. *Anal. Chem.* **2016**, *88*, 10266-10274.

(2) Zhang, Z. M.; Chen, S.; Liang, Y. Z. *Analyst* **2010**, *135*, 1138-1146.

(3) Cleveland, W. S.; Devlin, S. J. *J. Am. Stat. Assoc.* **1988**, *83*, 596-610.

(4) Zhou, M.; Sharma, R.; Zhu, H.; Li, Z.; Li, J.; Wang, S.; Bisco, E.; Massey, J.; Pennington, A.; Sjoding, M.; Dickson, R. P.; Park, P.; Hyzy, R.; Napolitano, L.; Gillies, C. E.; Ward, K. R.; Fan, X. *Anal. Bioanal. Chem.* **2019**, *411*, 6435-6447.

(5) Lau, H. C.; Yu, J. B.; Lee, H. W.; Huh, J. S.; Lim, J. O. *Sensors* **2017**, *17*, 1783.

(6) Wang, C.; Feng, Y.; Wang, M.; Pi, X.; Tong, H.; Wang, Y.; Zhu, L.; Li, E. *Sci. Rep.* **2015**, *5*, 1-9.

(7) Wang, C.; Li, M.; Jiang, H.; Tong, H.; Feng, Y.; Wang, Y.; Pi, X.; Guo, L.; Nie, M.; Feng, H.; Li, E. *Sci. Rep.* **2016**, *6*, 1-7.

(8) Smolinska, A.; Hauschild, A. C.; Fijten, R. R. R.; Dallinga, J. W.; Baumbach, J.; Van Schooten, F. J. *J. Breath Res.* **2014**, *8*, 027105.

(9) Smolinska, A.; Klaassen, E. M. M.; Dallinga, J. W.; Van De Kant, K. D. G.; Jobsis, Q.; Moonen, E. J. C.; Van Schayck, O. C. P.; Dompeling, E.; Van Schooten, F. J. *PLoS ONE* **2014**, *9*, e95668.

(10) Pereira, J.; Porto-Figueira, P.; Cavaco, C.; Taunk, K.; Rapole, S.; Dhakne, R.; Nagarajaram, H.; Câmara, J. S. *Metabolites* **2015**, *5*, 3-55.

(11) Morris, J. S.; Coombes, K. R.; Koomen, J.; Baggerly, K. A.; Kobayashi, R. *Bioinformatics* **2005**, *21*, 1764-1775.

(12) Nielsen, N. P. V.; Carstensen, J. M.; Smedsgaard, J. *J. Chromatogr. A* **1998**, *805*, 17-35.

(13) Tomasi, G.; Van Den Berg, F.; Andersson, C. *J. Chemom.* **2004**, *18*, 231-241.
